# Experimental evaluation of the GE NM/CT 870 CZT clinical SPECT system equipped with WEHR and MEHRS collimator

**DOI:** 10.1002/acm2.13158

**Published:** 2021-01-10

**Authors:** Toshimune Ito, Yohji Matsusaka, Masahisa Onoguchi, Hajime Ichikawa, Koichi Okuda, Takayuki Shibutani, Masaaki Shishido, Kozo Sato

**Affiliations:** ^1^ Department of Radiology Saiseikai Yokohamashi Tobu Hospital Yokohama Japan; ^2^ Department of Diagnostic Radiology School of Medicine Keio University Tokyo Japan; ^3^ Department of Quantum Medical Technology Graduate School of Medical Sciences Kanazawa University Kanazawa Japan; ^4^ Department of Radiology Toyohashi Municipal Hospital Toyohashi Japan; ^5^ Department of Physics Kanazawa Medical University Kahoku, Ishikawa Japan; ^6^ Department of Diagnostic Radiology Saiseikai Yokohamashi Tobu Hospital Yokohama Kanagawa Japan

**Keywords:** Discovery NM/CT 870 CZT ⇒ NM/CT 870 CZT, medium‐energy high‐resolution sensitivity collimator, wide‐energy high‐resolution collimator

## Abstract

**Purpose:**

A high‐energy‐resolution whole‐body SPECT‐CT device (NM/CT 870 CZT; C‐SPECT) equipped with a CZT detector has been developed and is being used clinically. A MEHRS collimator has also been developed recently, with an expected improvement in imaging accuracy using medium‐energy radionuclides. The objective of this study was to compare and analyze the accuracies of the following devices: a WEHR collimator and the MEHRS collimator installed on a C‐SPECT, and a NaI scintillation detector‐equipped Anger‐type SPECT (A‐SPECT) scanner, with a LEHR and LMEGP.

**Methods:**

A line phantom was used to measure the energy resolutions including collimator characteristics in the planar acquisition of each device using ^99m^Tc and ^123^I. We also measured the system's sensitivity and high‐contrast resolution using a lead bar phantom. We evaluated SPECT spatial resolution, high‐contrast resolution, radioactivity concentration linearity, and homogeneity, using a basic performance evaluation phantom. In addition, the effect of scatter correction was evaluated by varying the sub window (SW) employed for scattering correction.

**Results:**

The energy resolution with ^99m^Tc was 5.6% in C‐SPECT with WEHR and 9.9% in A‐SPECT with LEHR. Using 123I, the results were 9.1% in C‐SPECT with WEHR, 5.5% in C‐SPECT with MEHRS, and 10.4% in A‐SPECT with LMEGP. The planar spatial resolution was similar under all conditions, but C‐SPECT performed better in SPECT acquisition. High‐contrast resolution was improved in C‐SPECT under planar condition and SPECT. The sensitivity and homogeneity were improved by setting the SW for scattering correction to 3% of the main peak in C‐SPECT.

**Conclusion:**

C‐SPECT demonstrates excellent energy resolution and improved high‐contrast resolution for each radionuclide. In addition, when using 123I, careful attention should be paid to SW for scatter correction. By setting the appropriate SW, C‐SPECT with MEHRS has an excellent scattered ray removal effect, and highly homogenous imaging is possible while maintaining the high‐contrast resolution.

## 1 INTRODUCTION

Recent studies have reported the effectiveness of a gamma camera equipped with a cadmium‐zinc‐telluride (CZT) detector. Moreover, a device that was developed specifically for the heart has been used clinically mainly in the field of cardiology.^1^, ^2^, ^3^, ^4^, ^5^, ^6^, ^7^, ^8^ Upon application of high voltage to the electrodes sandwiching the crystal, the CZT detector measures the electric charge by collecting the generated electrons/holes when radiation enters the crystal and interacts with it. The energy required to generate an electron–hole pair is about 5 eV; this energy can be detected by various sensors and precisely measured, yielding an energy resolution higher than that of the Anger‐type detector.^3^ In addition, because the band gap in the CZT detector is large, it can be used at room temperature, and the high atomic number of CZT assists in efficient photoelectric absorption, leading to an improved system sensitivity compared with that of the Anger‐type detector. Thus, broad clinical applications of CZT‐based imaging can be expected. Therefore, a whole‐body single‐photon emission computed tomography (SPECT)–computed tomography (CT) device using a CZT detector (C‐SPECT) with these excellent system characteristics was developed. This device consists of a two‐detector SPECT scanner and a 16‐row CT scanner. Thirteen 4‐cm × 4‐cm CZT units are arranged in each detector in the *x*‐direction and 10 in the y‐direction. This device has an effective visual field of 51 cm × 39 cm. In addition, as compared with a conventional NaI scintillation detector‐equipped Anger‐type SPECT (A‐SPECT), the improved contrast resolution afforded by higher energy resolution of this pixel‐type detector may be effective in the clinical setting. Clinical studies using this device have included the verification of dual isotope imaging and short time acquisition using ^99m^Tc and ^123^I in myocardial blood flow tests.^9^, ^10^ However, no studies have focused on the evaluation of the performance of the device itself. In addition to the wide‐energy high‐resolution (WEHR) collimator that is standard for this device, a medium‐energy high‐resolution sensitivity (MEHRS) collimator was recently developed. This collimator is expected to improve imaging accuracy of medium‐energy radionuclides. Thus, verifying the performance of this device in clinical applications is of utmost importance. In this study, we analyzed the system performance of the C‐SPECT device using ^99m^Tc (a low‐energy radionuclide) and ^123^I (a low‐ and medium‐energy radionuclide), both of which are widely used in clinical practice.^11^ The system performance assuming clinical indications was evaluated by comparing the device with a two‐detector SPECT equipped with an NaI scintillation detector.

## 2 EQUIPMENT/METHOD

### 2.A Acquisition of the device/data‐processing device

**Fig. 1:**
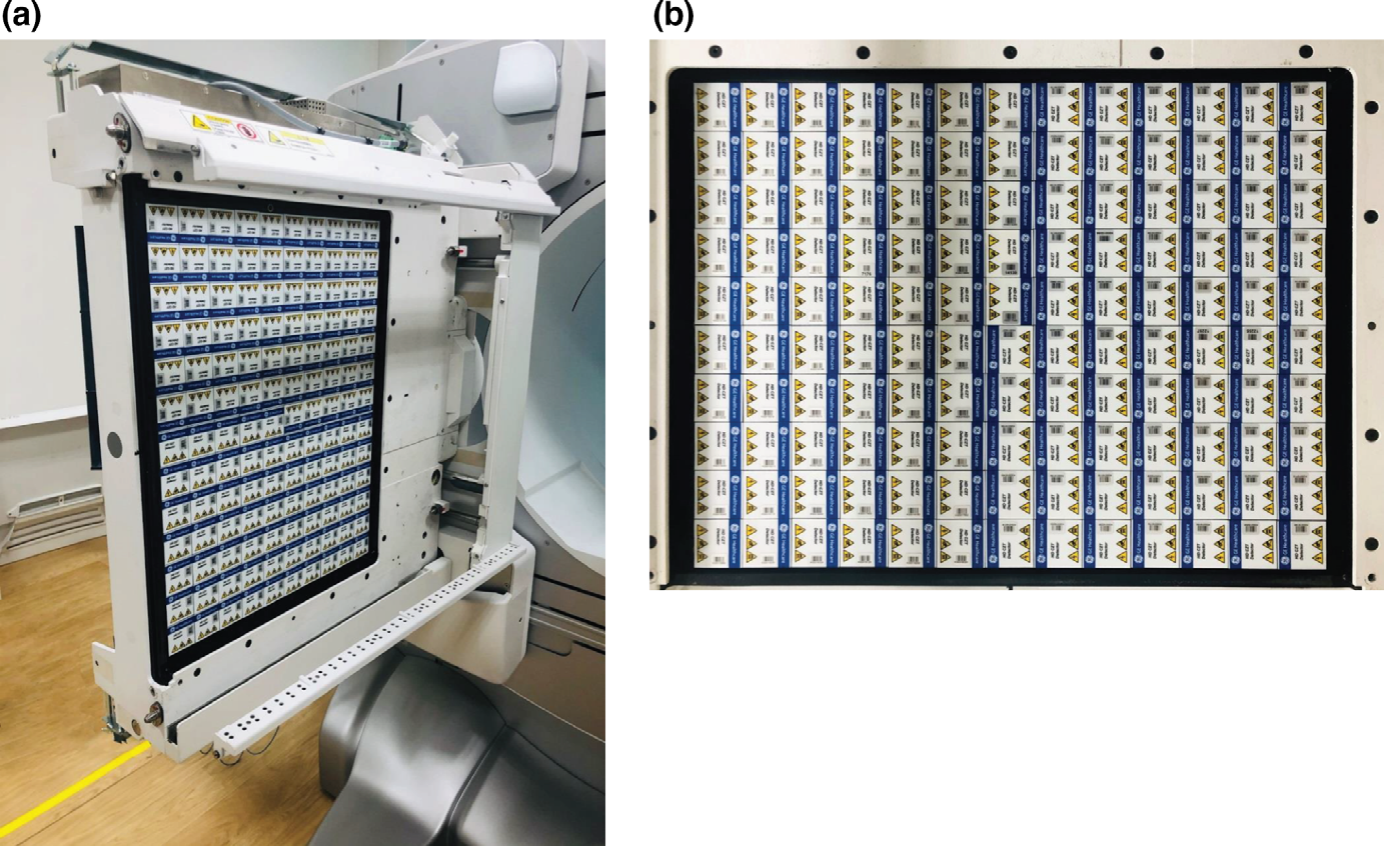
(a) External appearance of a CZT detector. (b) Detector arrangement. There are 13 detectors placed in the *x*‐direction and 10 in the *y* direction.

We used the NM/CT 870 CZT device equipped with a whole‐body CZT detector (GE Healthcare, Waukesha, WI, USA; Fig. 1), and the E.CAM device equipped with an NaI scintillation detector (Canon Medical Systems Tokyo, Japan). WEHR (C‐SPECT_WEHR) and MEHRS (C‐SPECT_MEHRS) were used as collimators for the C‐SPECT device. A low‐energy high‐resolution (LEHR) collimator (A‐SPECT_LEHR) for ^99m^Tc and a low‐medium‐energy general purpose (LMEGP) collimator (A‐SPECT_LMEGP) for ^123^I were used on the A‐SPECT device, while keeping clinical practice in mind. The design parameters of the WEHR, MEHRS, LEHR, and LMEGP collimators are shown in Table 1. Although the parameters of LMEGP were not disclosed, the system spatial resolution and sensitivity were LEHR × 0.70 and LEHR × 1.71 at the manufacturing stage respectively. Xeleris 4.0 (GE Healthcare) and E‐Soft (Canon Medical Systems) were used as image‐processing devices. For image analysis, we used the general image‐processing software Prominence Processor Version 3.1^12^ (Nihon Medi‐Physics Co Ltd, Tokyo, Japan), ImageJ (National Institutes of Health, MD, USA), and Demon Research Image Processor Version 3.01 (FUJIFILM Toyama Chemical Co., Tokyo, Japan). Radionuclide, ^99m^Tc‐incardronate (Nihon Medi‐Physics Co Ltd, Tokyo, Japan) and ^123^I‐IMP perfuzamine (Nihon Medi‐Physics Co Ltd, Tokyo, Japan).

**Table 1:** Collimator designs.

	Type of hole	Hole length (mm)	Hole diameter (mm)	Septal thickness (mm)	Number of holes	penetration (%)
LEHR	Hexagonal	24.05	1.11	0.16	148000	1.5 (^99m^Tc)
WEHR	Square	45	2.26	0.2	33280	0.55 (^99m^Tc)
LMEGP	Undisclosed	Undisclosed	Undisclosed	Undisclosed	Undisclosed	< 0.25 (^123^I)
MEHRS	Hexagonal	40.25	2.8	0.9	Undisclosed	1.8 (^111^In)
						0.3 (^177^Lu)

#### 2.B Planar acquisition

Energy resolution, spatial resolution, and system sensitivity measurements were performed using a method that conformed to the NEMA standards^13^ to the best possible extent.

### 2.B.1 Energy resolution

We sealed 84.1 (MBq /ml) of ^99m^Tc and 87.7 (MBq /ml) of ^123^I in a self‐made line phantom with a diameter of 1.5 mm and length of 30.0 cm. For C‐SPECT_WEHR and A‐SPECT_LEHR with ^99m^Tc, and for C‐SPECT_WEHR, C‐SPECT_MEHRS, and A‐SPECT_LMEGP with ^123^I, planar images were acquired to achieve 10 kilo counts (kct) at 140 and 159 keV peak energy for ^99m^Tc and ^123^I respectively. Furthermore, the counting rate was set to ≤20 kct/s. Acquisitions of two placements of the line phantom were performed: one without a scatterer (scatter (−)), and one with a water‐equivalent phantom (Kyoto Kagaku Co., Kyoto, Japan) placed 5 cm behind and 10 cm in front of the line phantom (scatter (+)). The conditions of data acquisition were as follows: C‐SPECT was performed in a 512 × 512 matrix at 2 × magnification with a pixel size of 0.55 mm. A‐SPECT was performed at 2 × magnification in a 512 × 512 matrix with a pixel size of 0.59 mm. The distance between the radiation source and the collimator was set to 10 cm. From the obtained γ‐ray photopeak energy spectrum, we calculated the energy resolution, including the collimator characteristics using the following Eq. (1), the photopeak energy Ep (^99m^Tc: 140 keV, ^123^I:159 keV), and the peak width Δ Ep (full‐width half‐maximum; FWHM), equivalent to half the count at the peak.

(1) $$\text{Energy resolution}\;=\;\left(\Delta \text{Ep}/\text{Ep}\right)\;\times 100\left(\%\right)$$

### 2.B.2 Spatial resolution

In addition to the 1.5‐mm‐diameter line phantom, we also obtained planar images of the self‐made line phantom with a diameter of 15 mm under the same conditions, so that the count at each energy peak kev was set at 125 kct. The conditions of data acquisition were as follows: for ^99m^Tc, C‐SPECT_WEHR was performed in a 512 × 512 matrix at 2 × magnification with a pixel size of 0.55 mm and in a 256 × 256 matrix at 0.898 × magnification with pixel size of 2.46 mm (native pixel). The energy window (EW) was set at 10% (C‐SPECT_WEHR10) and 15% (C‐SPECT_WEHR15). A‐SPECT_LEHR was performed at 2 × magnification in a 512 × 512 matrix with a pixel size of 0.59 mm and at 1 × magnification in a 256 × 256 matrix with a 2.40‐mm pixel size and EW of 15%. The pixel size in A‐SPECT was set to be the closest to C‐SPECT. For ^123^I, an EW of 15% was set under the same conditions as for ^99m^Tc for each collimator. Next, using the data obtained, 10 rows from the center under each acquisition condition of the line phantoms were added, and the FWHM was calculated from the profile curve.

### 2.B.3 System sensitivity

Aqueous solutions of 196 MBq ^99m^Tc and 183 MBq ^123^I were sealed in a circular container with a diameter of 9 cm and a height of 0.9 cm. Each collimator was installed, and data were acquired with a radiation source‐collimator distance of 100 mm and a total count (Ct) of 4000 kct. The conditions of data acquisition were 2 × magnification with a 512 × 512 matrix under each condition. Next, using the acquired data, we set a rectangular region of interest (ROI) surrounding the entire image. We then measured the Ct of the entire image and calculated the in‐air system sensitivity, Ssys (count/s/MBq), from Eqs. (2) and (3).

(2) $$Rt=Ct\times exp\left[\frac{\left(T‐T_{\mathrm{cal}}\right)}{T_{\mathrm{half}}}ln2\right]\times \left[\frac{ln2}{T_{\mathrm{half}}}\right]{\left[1‐exp\left[‐\frac{T_{\mathrm{acq}}}{T_{\mathrm{half}}}ln2\right]\right]}^{‐1}$$

(3) $$S_{\mathrm{sys}}=\frac{Rt_{100}}{A_{\mathrm{cal}}}$$

Here, Rt is the count rate corrected for radioactive decay (cps), T is the acquisition start time, T_acq_ is the acquisition time (seconds), T_cal_ is the radioactivity measurement time of the dose calibrator, T_half_ is the half‐life of ^99m^Tc (21,654 s) or ^123^I (47,772 s), and A_cal_ is the radioactivity (MBq) measured by the dose calibrator.

### 2.B.4 High‐contrast resolution

With the C‐SPECT_WEHR and A‐SPECT_LEHR installed, a plane phantom with lead bars of 2.0, 2.5, 3.0, and 3.5‐mm width and spacing was fixed to a plane source containing 370 MBq of ^99m^Tc. The distance between the collimator and bar phantom was fixed at 10 cm. Assuming clinical planar acquisition,^14^, ^15^ the counts were set at 500 kct and 1000 kct, with 6600 kct used as a reference from which statistical noise could be sufficiently removed. The data acquisition conditions were set to 512 × 512 at 1 × magnification for each device. The pixel size was set to 1.1 mm for C‐SPECT and 1.2 mm for A‐SPECT, and each EW was set at 15%. Next, from the data obtained, the profile lines of 27 bars at a lead bar spacing of 2.0 mm, 23 bars at 2.5 mm, 18 bars at 3.0 mm, and 15 bars at 3.5 mm were calculated. In addition, the reference image was subjected to two‐dimensional Fourier transformation for frequency evaluation. The radial‐directional intensity distribution function Pr(n) was calculated to conduct a one‐dimensional evaluation of the two‐dimensional frequency distribution under each condition.^16^

### 2.C SPECT acquisition

#### 2.C.1 SPECT resolution evaluation

A 1.5‐mm‐diameter line source was filled with 110 MBq/mL of ^99m^Tc. This source was placed in the center of a 25‐cm‐diameter cylindrical phantom (JSP, Kyoto Kagaku, Kyoto, Japan) that was filled with water as a scatterer and 10 cm away from the phantom center in the outer peripheral direction of the *x‐* and *y*‐axes. The data acquisition conditions for C‐SPECT_WEHR10 and C‐SPECT_WEHR15 were 128 × 128 matrix size, and 1.797 × magnification with a pixel size of 2.46 mm. For A‐SPECT_LEHR, the matrix size was 128 × 128 with a 2 × magnification, 2.40 mm pixel size, and EW at 15%. Acquisition was performed so that a rotational radius of 15 cm and a 3‐degree step of 120 views would result in one view of 100 kct. Next, 105 MBq/mL of ^123^I was sealed in the line source, and the phantom was placed under the same conditions. The data acquisition conditions were the same as ^99m^Tc for C‐SPECT_WEHR, C‐SPECT_MEHRS, and A‐SPECT_LMEGP, each with the EW set to 15%. Scatter correction (SC) and attenuation correction were not performed in each acquisition. Next, the obtained data were reconstructed by filter‐back projection, and the FWHM was calculated from the axial images by constructing a profile curve after adding six cross sections in both the radial and tangential directions. Furthermore, the aspect ratio (ASR) was calculated from Eq. (4) as a distortion evaluation of the obtained FWHM.

(4) $$\text{ASR}\;={\;\text{FWHM}}_{\text{radial}}/{\;\text{FWHM}}_{\text{tangential}}$$

#### 2.C.2 Radioactivity concentration linearity

^99m^Tc aqueous solutions of 370, 296, 222, 148, and 74 kBq/mL were sealed in the five columnar inserts of the radioactivity concentration linearity phantom (JSP, Kyoto Kagaku Co.).^17^, ^18^ Six aqueous solutions, one of which was filled only with water, were positioned, and a 37‐kBq/mL ^99m^Tc aqueous solution was sealed in the surrounding as the background. Under the same conditions as in the spatial resolution evaluation, data were acquired for a total time of 10, 20, 30, 40, 50, and 60 min.

As SC of C‐SPECT, the lower sub window (SW) of 7% (119.7keV – 129.5keV) in ^99m^Tc (^99m^Tc_SC7) and the lower SW of 7% (136.0keV – 147.1keV) and upper SW of 7% (170.9keV – 182.0keV) in ^123^I (^123^I_SC7) were also acquired with respect to the main peak. Furthermore, the lower SW of 3% (125.3keV – 129.5keV) in ^99m^Tc (^99m^Tc_SC3) and the lower SW of 3% (142.3keV – 147.1keV) and upper SW of 3% (170.9keV – 175.7keV) in ^123^I (^123^I_SC3) were also acquired with respect to the main peak. As SC of A‐SPECT, ^99m^Tc_SC7 and ^123^I_SC7 were also acquired with respect to the main peak.

Next, the six sealed pieces in the columnar part were filled with ^123^I aqueous solutions of 80, 40, 20, 10, and 5 kBq/mL,^19^, ^20^ as well as one with only water. The surrounding volume was sealed with a 2.5 kBq/mL ^123^I aqueous solution as the background. Data were collected under the same conditions as for ^99m^Tc, with a total time of 20, 40, 60, 80, 100, and 120 min. Next, the obtained data were reconstructed by filter‐back projection. Pre–Butterworth filter processing (cutoff of 0.60 cycle/cm, order 8) was used for smoothing processing. The following were performed as corrections: attenuation correction by the Chang method was performed using a linear attenuation coefficient of 0.150 cm^−1^ in ^99m^Tc and 0.120 cm^−1^ in ^123^I. SC was performed using the dual energy window (DEW) method^21^ in ^99m^Tc, where scattered rays are estimated and subtracted by multiplying a scale factor of 0.5 to the count obtained by a low‐energy side setting. For ^123^I, SC was performed via the triple energy window (TEW) method.^22^ From the reconstructed axial images, a circular ROI was set at 70% of the inner diameter in each cylinder. The Ct value was measured, and the regression line with the sealed radioactivity concentration was calculated using the least‐squares method. Moreover, we calculated the following: the scattered ray content rate obtained by the intercept and the coefficient of determination (*R* ^2^) in order to evaluate the accuracy of the regression line.

#### 2.C.3 Contrast resolution

A 74‐mBq/mL ^99m^Tc aqueous solution was sealed in a Hot‐Rod‐type phantom with diameters of 4.0, 6.0, 8.0, 10.0, 12.0, and 15.0 mm (JSP, Kyoto Kagaku Co.). The data were collected for 30 min under the same conditions as that of the spatial resolution evaluation. Next, a 44‐kBq/mL ^123^I aqueous solution was sealed, and the data were acquired for 60 min under the same conditions as those for ^99m^Tc. Next, the obtained data were reconstructed in the same manner as used for the evaluation of the radioactivity concentration linearity. Furthermore, Pr(n) was calculated from the reconstructed axial image.

#### 2.C.4 Homogeneity

A 74‐mBq/mL ^99m^Tc aqueous solution was sealed in 25‐cm‐diameter cylindrical phantom. Reconstruction was performed under the same condition as the radioactivity concentration linearity evaluation. A circular ROI was set at 70% of the inner diameter on the 10 axial image slices of the phantom center. The average value of the percentage coefficient of variation (%CV) of 10 images was calculated from Eq. (5). The same process was then repeated with a sealed 44‐kBq/mL ^123^I aqueous solution. Note that Ct is the average count value within the ROI, and SD is the standard deviation within the ROI.

(5) %CV=SD/Ct×100

## 3 RESULTS

### 3.A Planar acquisition

**Fig. 2:**
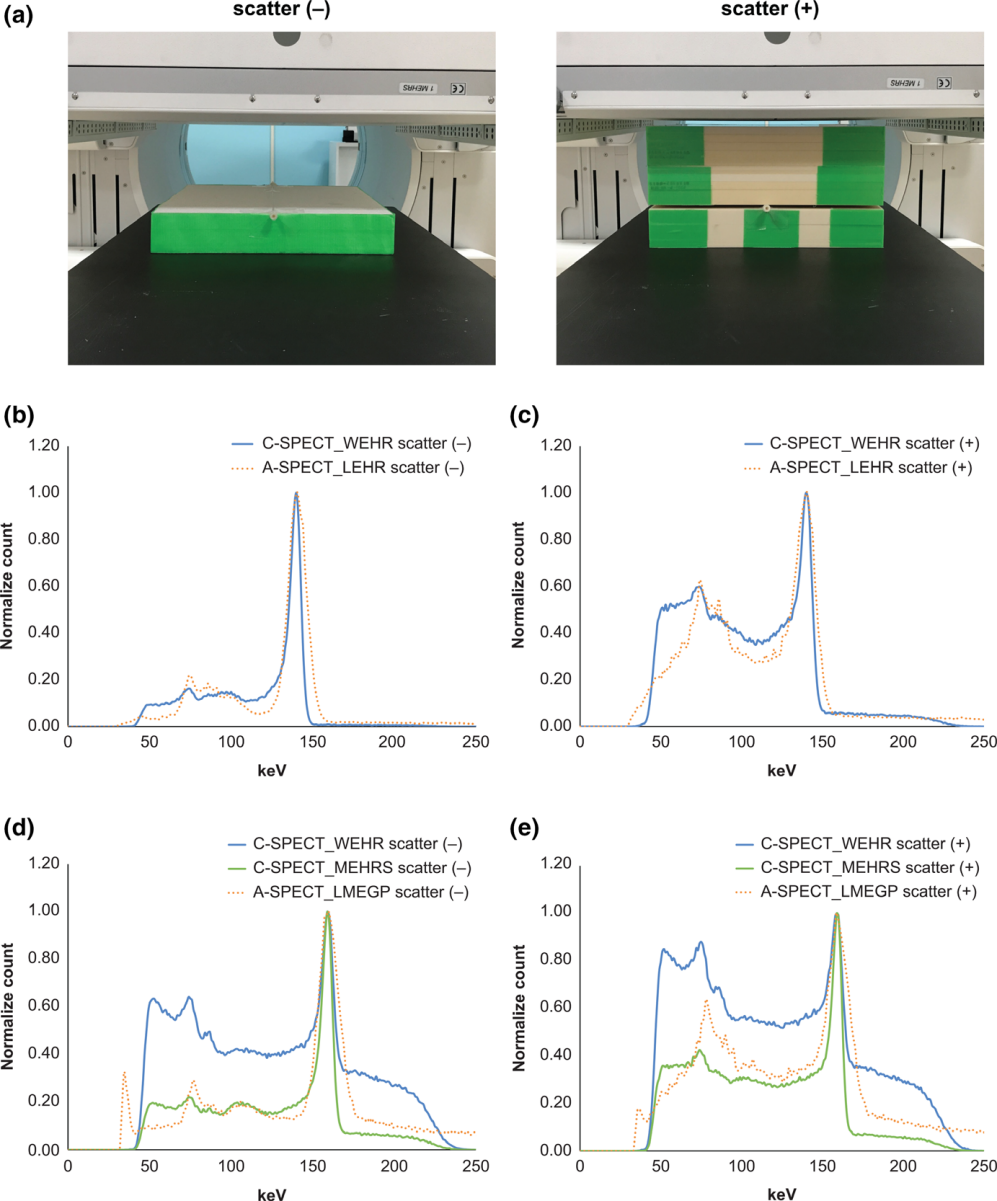
Energy spectrum of the system environment of each radionuclide. (a) Line phantom set up with the GE Discovery 870 CZT SPECT‐CT system. (b) Energy spectrum of scatter (−) of each device with each collimator installed in the ^99m^Tc formulation. (c) Energy spectrum of scatter (+) of each device with each collimator installed in the ^99m^Tc formulation. (d) Energy spectrum of scatter (−) of each device with each collimator installed in the ^123^I formulation. (e) Energy spectrum of scatter (+) of each device with each collimator installed in the ^123^I formulation.

**Fig. 3:**
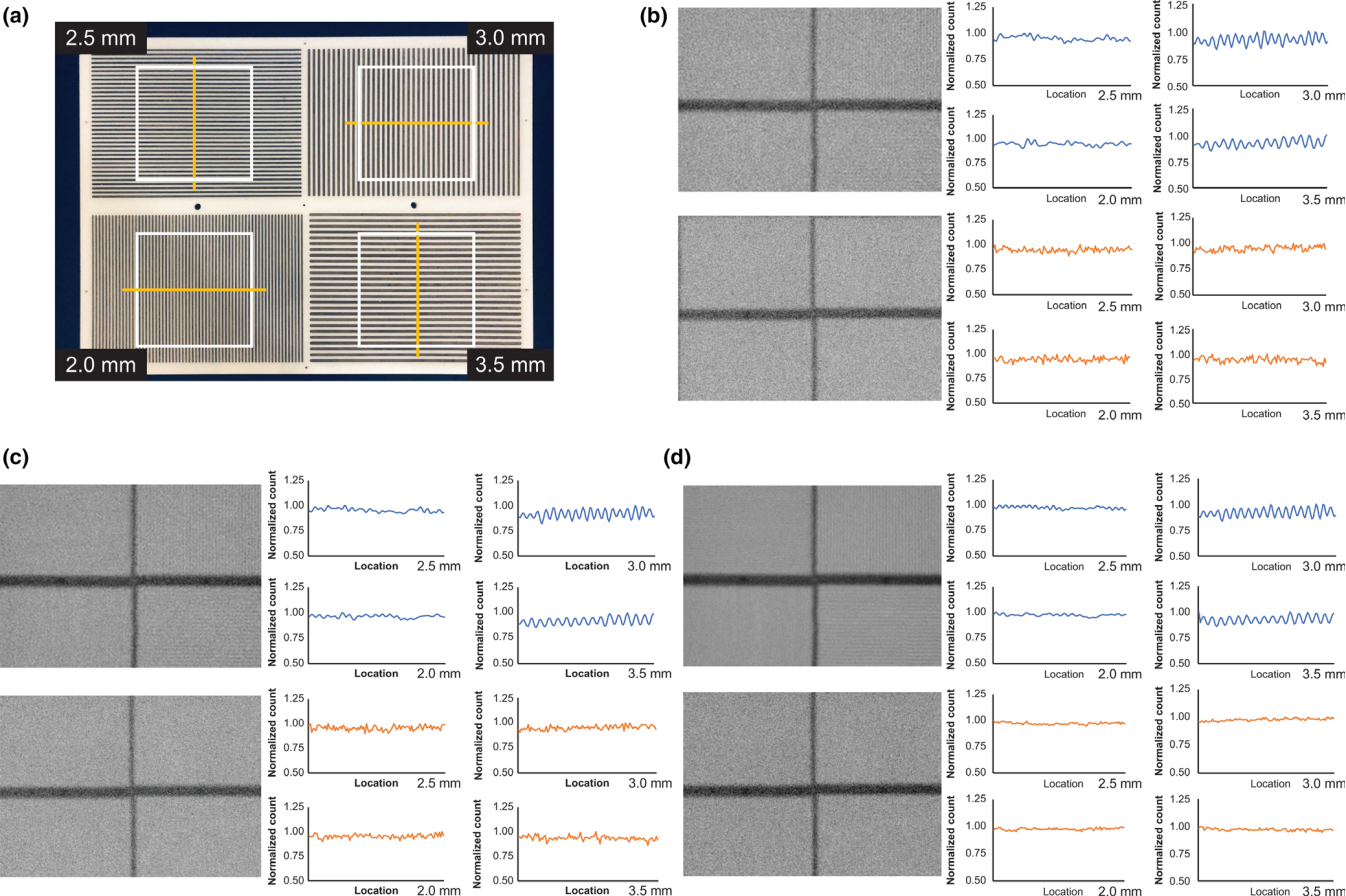
Images and count profile curve for the evaluation of contrast resolution in planar acquisition. (a) Bar phantom image and profile range. (b) Ct: 500 kilo counts (kct). (c) Ct: 1000 kct. (d) Ct: 6600 kct. [Upper row of figure (b, c, d)] C‐SPECT with WEHR collimator, [Lower row of figure (b, c, d)] A‐SPECT with LEHR collimator.

**Fig. 4:**
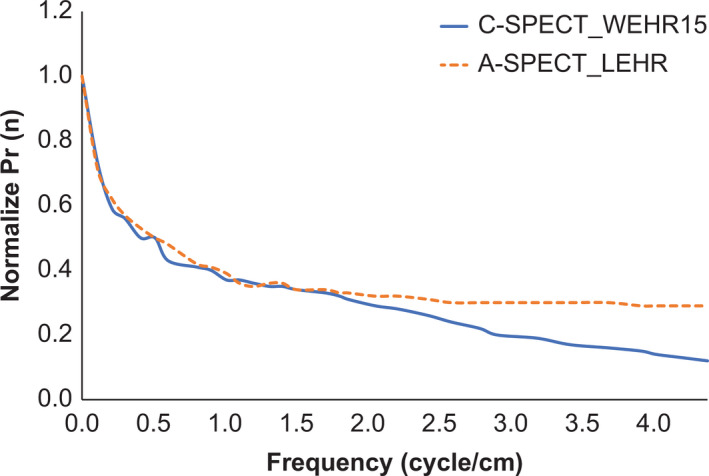
Radial‐directional intensity distribution function Pr(n) in a bar phantom image with Ct 6600 kct with ^99m^Tc formulation.

Figure 2 shows the energy spectrum for each device and collimator. C‐SPECT_WEHR had high relative counts in the Compton region for each radionuclide. With ^123^I, the tendency was the strongest for C‐SPECT_WEHR, but the relative count in the Compton region was greatly reduced for C‐SPECT_MEHRS and was lower than that for A‐SPECT _LMEGP. Moreover, the same tendency was observed with the inclusion of scatterers. With ^99m^Tc, the energy resolution was 5.6% for C‐SPECT_WEHR and 9.9% for A‐SPECT_LEHR. With ^123^I, it was 9.1% for C‐SPECT_WEHR, 5.5% for C‐SPECT_MEHRS, and 10.4% for A‐SPECT_LMEGP. Table 2 shows the results of the spatial resolution evaluation. For the line phantom with a diameter of 1.5 mm using ^99m^Tc, the FWHM showed the lowest value for A‐SPECT pixel size of 0.59 mm, while those for pixel size of A‐SPECT 2.40 mm and all the conditions of C‐SPECT were the same. The same tendency was observed for the diameter 15 mm line phantom. With ^123^I, the FWHM of C‐SPECT_WEHR had the lowest value. Moreover, A‐SPECT_LMEGP had the lowest resolution. Furthermore, the FWHM was the same under each condition for both pixel sizes. The same tendency was observed for the 15‐mm diameter line phantom. For the evaluation of system sensitivity, Table 3 shows that with ^99m^Tc, the value was highest in A‐SPECT_LEHR and lowest in C‐SPECT_WEHR10. With ^123^I, A‐SPECT_LMEGP and C‐SPECT_WEHR were equivalent to each other and higher than C‐SPECT_MEHRS. Figure 3 shows the bar phantom image and line profile of each count as a high‐contrast resolution evaluation. In C‐SPECT at 500 and 1000 kct, a bar of 3.0 mm matched the actual number of bars with the number of count peaks shown in the line profile. A‐SPECT was inseparable for all bar widths. In addition, from the frequency evaluation, C‐SPECT_WEHR15 showed falling signal strength from about 1.60 cycles/cm onward (Fig. 4). However, A‐SPECT_LEHR showed a constant signal strength from 1.60 cycles/cm onward.

**Table 2:** Results of FWHM in planar acquisition under each system. (a) ^99m^Tc formulation; (b) ^123^I formulation.

	C‐SPECT_WEHR10		C‐SPECT_WEHR15		A‐SPECT_LEHR	
(a)						
mm/pixel	0.59	2.46	0.59	2.46	0.55	2.39
Line (1.5 mm)	7.9	7.9	7.8	7.9	7.2	7.9
Line (15 mm)	11.8	11.8	11.7	11.7	11.3	11.7

	C‐SPECT_WEHR		C‐SPECT_MEHRS		A‐SPECT_LMEGP	
(b)						
mm/pixel	0.59	2.46	0.59	2.46	0.55	2.39
Line (1.5 mm)	7.9	7.9	9.8	9.8	10.4	10.7
Line (15 mm)	11.5	11.6	12.6	12.6	13.1	13.2

**Table 3:** Results of system sensitivity. (a) ^99m^Tc formulation; (b) ^123^I formulation.

	C‐SPECT_WEHR10	C‐SPECT_WEHR15	A‐SPECT_LEHR
(a)			
S_sys_ (cps/MBq)	69	78	88

	C‐SPECT_WEHR	C‐SPECT_MEHRS	A‐SPECT_LMEGP
(b)			
S_sys_ (cps/MBq)	117	74	116

### 3.B SPECT acquisition

**Fig. 5:**
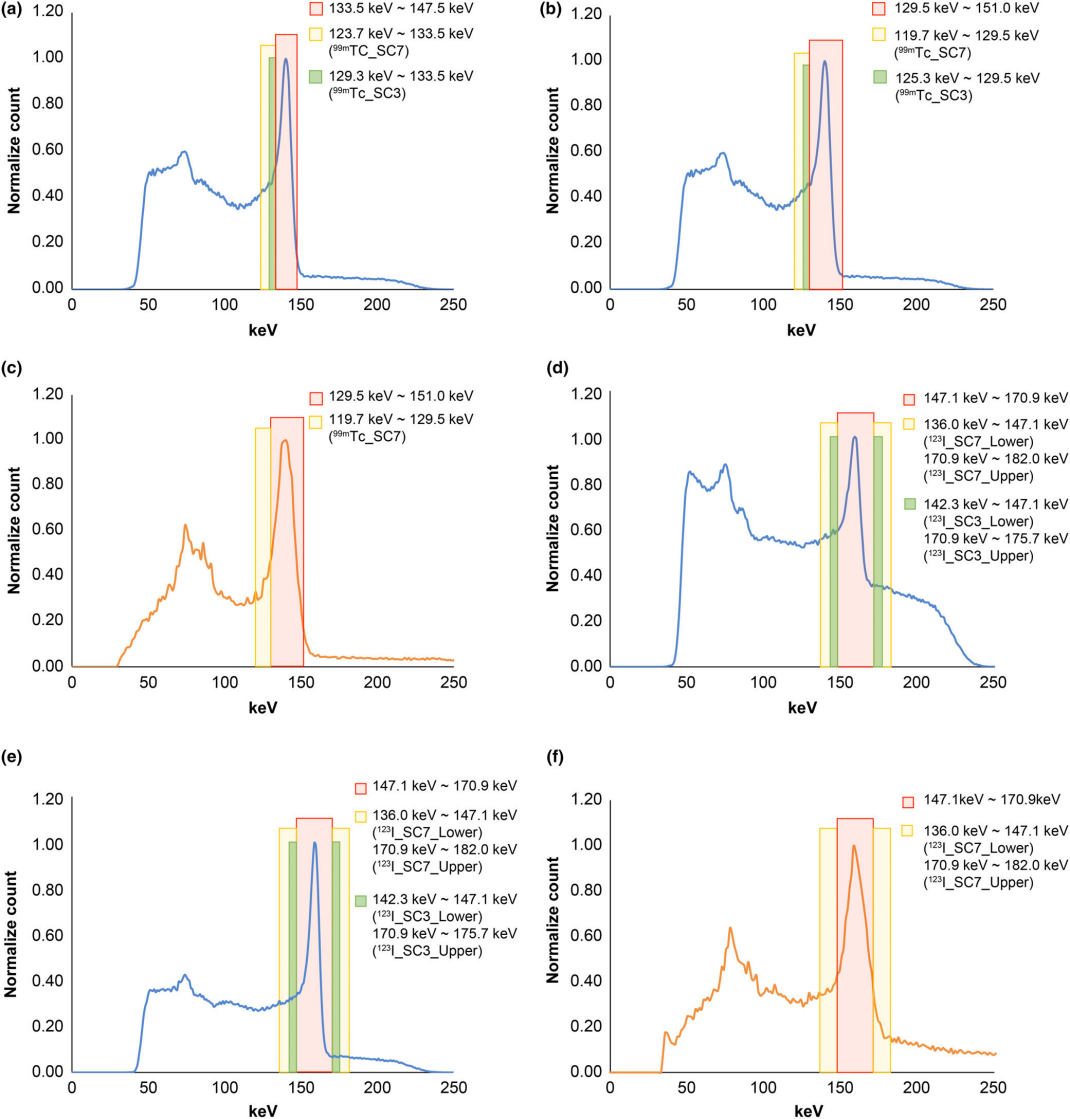
Energy spectrum of device primary and scattered photon windows with each collimator installed. (a) C‐SPECT_WEHR10 for ^99m^Tc formulation. (b) C‐SPECT_WEHR15 for ^99m^Tc formulation. (c) A‐SPECT_LEHR for ^99m^Tc formulation. (d) C‐SPECT_WEHR for ^123^I formulation. (e) C‐SPECT_MEHRS for ^123^I formulation. (f) A‐SPECT_LMEGP for ^123^I formulation.

**Fig. 6:**
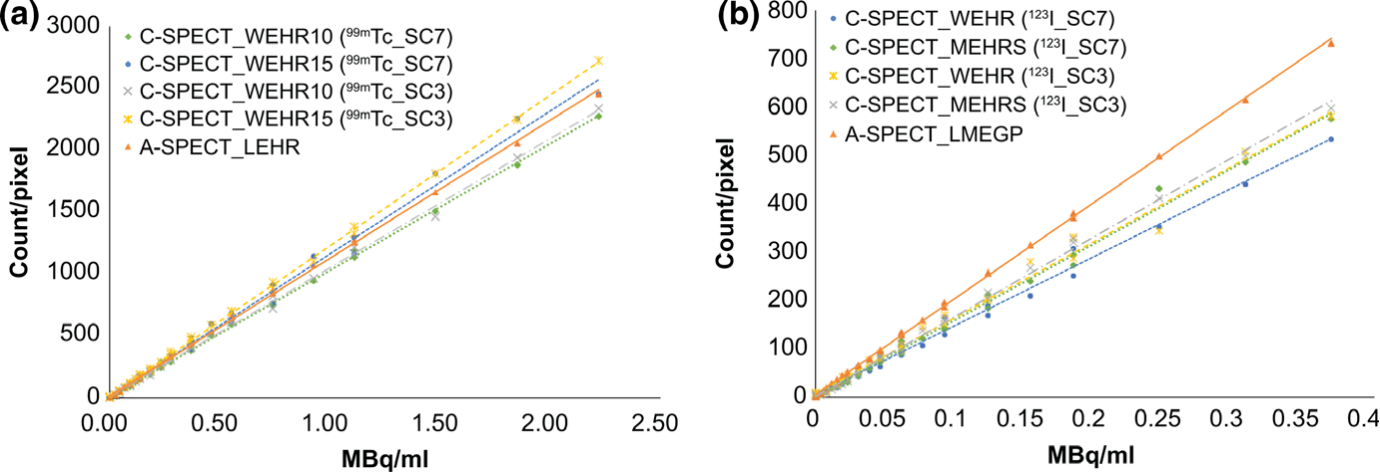
Regression formula of radioactivity concentration linearity in each system. (a) ^99m^Tc formulation; (b) ^123^I formulation.

Table 4 shows the results of the SPECT spatial resolution evaluation. Similar results were obtained under each condition with ^99m^Tc. With ^123^I, C‐SPECT_WEHR had the highest spatial resolution, and the results were comparable between C‐SPECT_MEHRS and A‐SPECT_LMEGP. With respect to ASR, there smaller distortions in A‐SPECT than in C‐SPECT between the *x*‐ and *y*‐axis peripheral lines. Figure 5 shows the energy spectrum along with primary and scattered photon windows of the device with each collimator and radionuclide. Figure 6 shows the regression line for the radioactivity concentration linearity evaluation and each radionuclide, collimator and energy window combination. With ^99m^Tc, the slope of C‐SPECT_WEHR15 was higher than that of A‐SPECT_LEHR and it was lowest in C‐SPECT_WEHR10. The scatter fraction was the lowest for ^99m^Tc_SC7 of C‐SPECT_WEHR10 and the highest for A‐SPECT^_^LEHR (Table 5). With ^123^I, the slope was the highest in A‐SPECT_LMEGP and lowest in ^99m^Tc_SC7 of C‐SPECT_WEHR. The scatter fraction was lowest in C‐SPECT_MEHRS and highest in C‐SPECT_WEHR (Table 5). Furthermore, ^123^I_SC3 of C‐SPECT_WEHR showed the highest value. The coefficient of determination of the regression line was close to 1.0 under all conditions (Table 5).

**Table 4:** Results of FWHM in SPECT acquisition under each system. (a) ^99m^Tc formulation; (b) ^123^I formulation.

FWHM	Center		X‐direction		Y‐direction	
	Tangential	Radial	Tangential	Radial	Tangential	Radial
(a)						
C‐SPECT_WEHR10	10.0	10.1	6.8	10.1	6.8	10.5
C‐SPECT_WEHR15	10.2	10.3	6.9	10.2	6.9	10.5
A‐SPECT_LEHR	10.1	10.0	7.6	10.1	7.7	10.0

	C‐SPECT_WEHR10	C‐SPECT_WEHR15	A‐SPECT_LEHR
FWHM (Average)	9.1	9.2	9.3

ASR	C‐SPECT_WEHR10	C‐SPECT_WEHR15	A‐SPECT_LEHR
Center	0.99	0.99	1.01
X‐direction	1.49	1.48	1.33
Y‐direction	1.54	1.52	1.30

FWHM	Center		x‐direction		y‐direction	
	Tangential	Radial	Tangential	Radial	Tangential	Radial
(b)						
C‐SPECT_WEHR	10.5	10.6	7.7	10.9	7.2	11.0
C‐SPECT_MEHRS	14.6	15.2	9.8	14.8	9.9	15.5
A‐SPECT_LMEGP	14.8	14.7	10.0	14.7	10.2	15.0

	C‐SPECT_WEHR	C‐SPECT_MEHRS	A‐SPECT_LMEGP
FWHM (Average)	9.7	13.3	13.2

Aspect ratio	C‐SPECT_WEHR	C‐SPECT_MEHRS	A‐SPECT_LMEGP
Center	0.99	0.96	1.01
*x*‐direction	1.42	1.51	1.48
*y*‐direction	1.59	1.57	1.46

**Table 5:** Results of concentration linearity in each system. (a) ^99m^Tc formulation; (b) ^123^I formulation.

	Slope	Intercept	R2	Scatter fraction (MBq/mL)
(a)				
C‐SPECT_WEHR10 (^99m^Tc_SC7)	1024	6.030	0.9994	0.0059
C‐SPECT_WEHR10 (^99m^Tc_SC3)	1044	7.877	0.9984	0.0075
C‐SPECT_WEHR15 (^99m^Tc_SC7)	1156	7.541	0.9991	0.0065
C‐SPECT_WEHR15 (^99m^Tc_SC3)	1219	9.429	0.9995	0.0077
A‐SPECT_LEHR	1117	11.61	0.9995	0.0104

	Slope	Intercept	R2	Scatter fraction (MBq/mL)
(b)				
C‐SPECT_WEHR (^123^I_SC7)	1433	4.897	0.9922	0.0034
C‐SPECT_WEHR (^123^I_SC3)	1579	6.642	0.9914	0.0042
C‐SPECT_MEHRS (^123^I_SC7)	1585	1.199	0.9952	0.0008
C‐SPECT_MEHRS (^123^I_SC3)	1656	1.437	0.9975	0.0009
A‐SPECT_LMEGP	1995	4.917	0.9995	0.0025

**Fig. 7:**
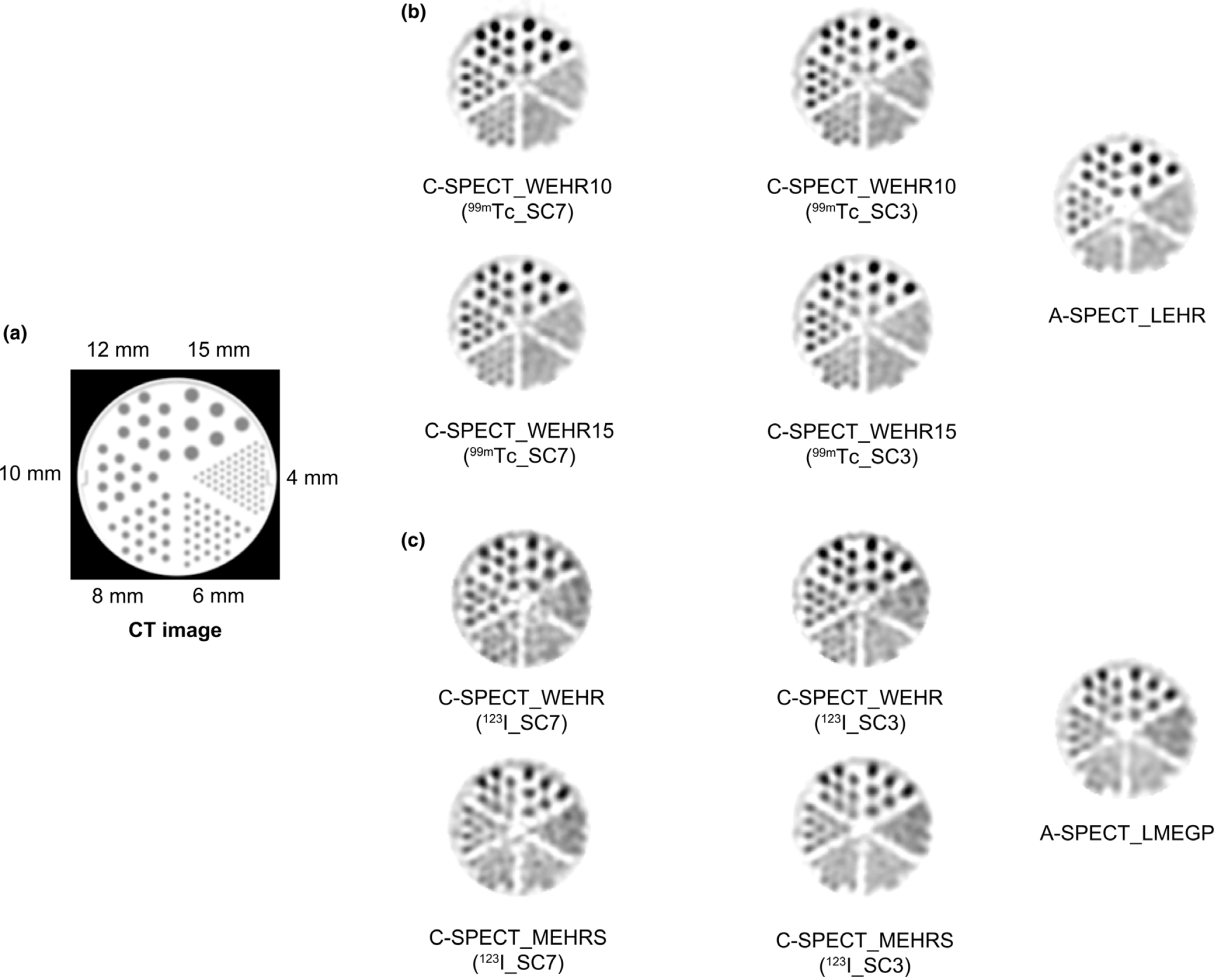
Image of the Hot‐Rod phantom in each system. (a) CT image; (b) ^99m^Tc formulation; and (c) ^123^I formulation.

**Fig. 8:**
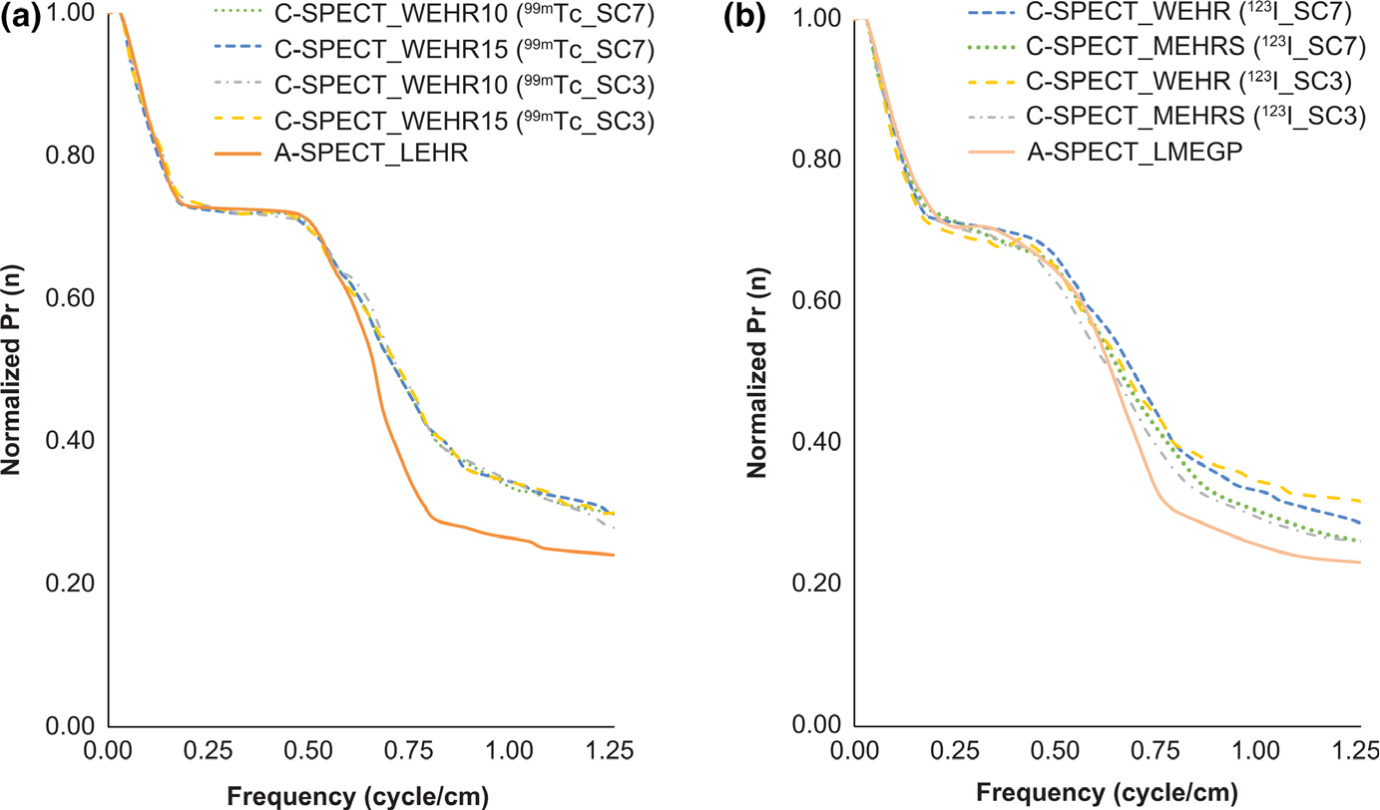
Radial‐directional intensity distribution function Pr(n) in Hot‐rod images from each system. (a) 99mTc formulation; (b) 123I formulation

For the evaluation of high‐contrast resolution, Fig. 7 shows the Hot‐Rod axial image, and Fig. 8 shows the Pr(n). Figure 8 shows that the signal strength was almost the same under all conditions up to the low‐frequency region, which is about the cutoff frequency of the Butterworth filter at 0.6 cycle/cm with ^99m^Tc. However, in subsequent higher‐frequency regions, C‐SPECT_WEHR showed higher signal strength than A‐SPECT_LEHR. There was no difference in signal strength between each EW and SW of C‐SPECT_WEHR. Similarly, ^123^I showed almost the same signal strength up to the low‐frequency region under all conditions, but in subsequent higher‐frequency regions, C‐SPECT_WEHR and C‐SPECT_MEHRS showed higher signal strengths than A‐SPECT_LMEGP, with the highest value seen in C‐SPECT_WEHR. The signal strength of ^123^I_SC3 was higher than that of ^123^I_SC7 in the high frequency range.

**Fig. 9:**
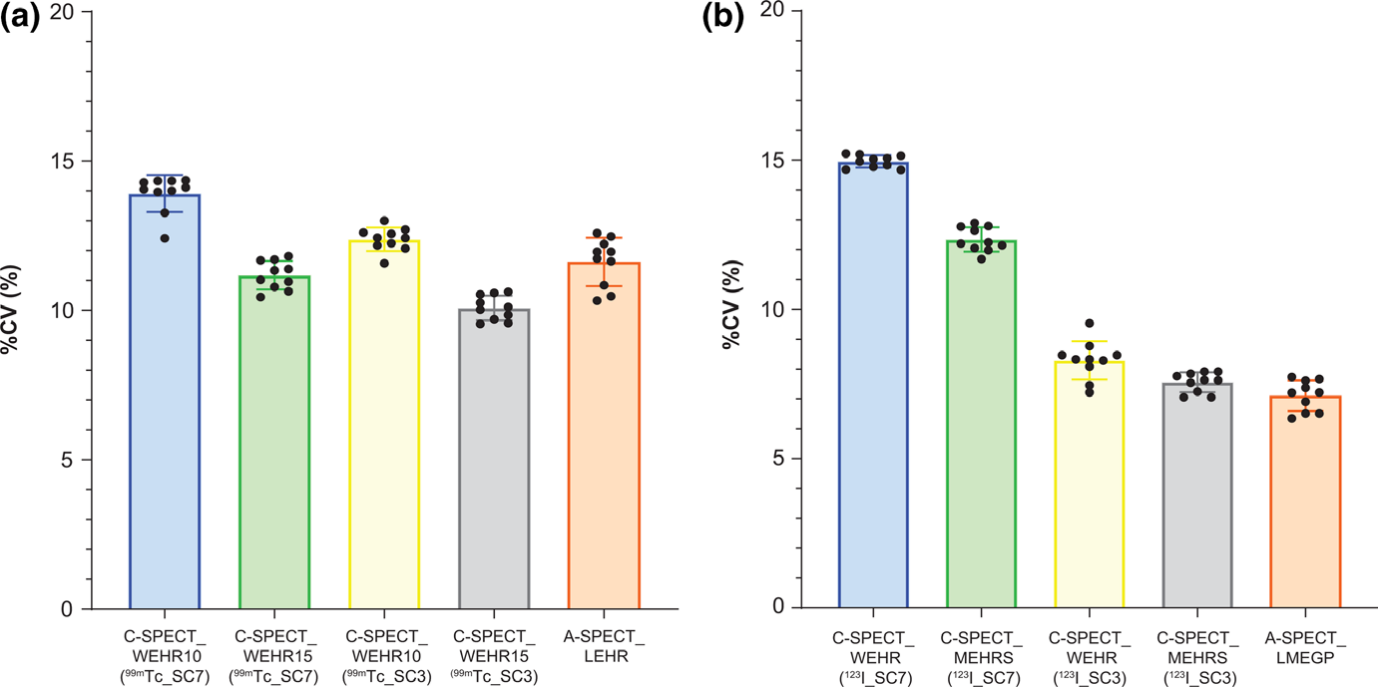
Results of the homogeneity in each system. (a) ^99m^Tc formulation; (b) ^123^I formulation.

Figure 9 shows the SPECT reconstructed homogeneity %CV of each radionuclide. For ^99m^Tc, the %CV values were 13.91% ± 0.62% in C‐SPECT_WEHR10 (^99m^Tc_SC7), 12.27% ± 0.39% in C‐SPECT_WEHR10 (^99m^Tc_SC3), 11.17% ± 0.48% in C‐SPECT_WEHR15 (^99m^Tc_SC7), 10.07% ± 0.41% in C‐SPECT_WEHR15 (^99m^Tc_SC3), and 11.62% ± 0.81% in A‐SPECT_LEHR. With ^123^I, C‐SPECT_WEHR (^123^I_SC7) was 14.95% ± 0.44%, C‐SPECT_WEHR (^123^I_SC3) was 8.29% ± 0.44%, C‐SPECT_MEHRS (^123^I_SC7) was 12.34% ± 0.41%, C‐SPECT_MEHRS (^123^I_SC3) was 7.56% ± 0.33%, and A‐SPECT_LMEGP was 7.10% ± 0.51%.

## 4 DISCUSSION

In this study, the performance of the C‐SPECT system was compared with that of A‐SPECT. The energy resolution of C‐SPECT_WEHR was improved by 1.75 times that of A‐SPECT_LEHR under the energy resolution evaluation using ^99m^Tc. This may be due to the following factors: (a) the characteristic of the CZT detector in which the threshold of the detector excitation is low and more signal energy can be acquired and (b) the collimator characteristic, in which one hole in the WEHR is geometrically equivalent to one of the pixel detectors. As such, it is possible to capture γ‐rays with high accuracy up to the edge of the detector (Fig. 1; Table 1). Next, in the evaluation using ^123^I, the energy resolution of C‐SPECT_MEHRS improved by 1.67 times that of C‐SPECT_WEHR and by 1.90 times that of A‐SPECT_LMEGP. This can be understood from the energy distribution in Fig. 2. First, the difference in the energy spectrum between the high‐energy region and the Compton region in WEHR and MEHRS in C‐SPECT (Fig. 2) is likely a result of the influence of 529 keV γ‐rays with ^123^I. The high‐energy γ‐rays of 529 keV penetrate the WEHR collimator more easily. The scattered rays derived from the obliquely entering 529‐keV γ‐rays are likely to enter the crystal. As such, the distribution of the scattered rays has a tendency to spread widely toward the high‐energy end. Additionally, the low‐energy scattered rays are derived from both the 529 and 159‐keV rays. Furthermore, in C‐SPECT, because of the incomplete charge acquisition of electron–hole pair and intercrystal scattering, the scattering component increases as a result of the effects of hole tailing^23^, ^24^, ^25^ in the Compton region. Therefore, MEHRS could derive a high‐energy resolution energy spectrum that suppresses the low‐energy and high‐energy scattering components by increasing the thickness of the septum and removing the scattering components compared to WEHR.

The system spatial resolution of the planar acquisition (Table 2) with ^99m^Tc was improved with a smaller pixel size in A‐SPECT. However, it did not change with the pixel size of 2.46 mm or less in C‐SPECT, regardless of EW. This is likely due to the fact that when the element size is 2.46 mm (native pixel) or smaller, a blur component is included to construct an image by interpolation processing in each element, and the spatial resolution deteriorates compared with A‐SPECT. Furthermore, images with pixel dimensions different from the native element dimensions must necessarily be interpolated. With ^123^I, C‐SPECT and A‐SPECT showed a similar tendency in relation to the pixel size. Furthermore, C‐SPECT_WEHR had a higher resolution than C‐SPECT_MEHRS. This is because WEHR is designed to have a larger hole length and a smaller hole diameter than MEHRS, resulting in improved spatial resolution.

Next, the evaluation of system sensitivity (Table 3) with ^99m^Tc showed that A‐SPECT_LEHR had a higher sensitivity than C‐SPECT_WEHR. The reason for this is that the LEHR is designed to have a smaller hole diameter than the WEHR for improved resolution, but to maintain acceptable sensitivity, the septa are designed to be thinner and the hole length is designed to be approximately 46% lower than WEHR (Table 1). For ^123^I, C‐SPECT_WEHR exhibited a higher sensitivity than C‐SPECT_MEHRS, which likely occurs because of the inclusion of more scatter from 529 keV septal penetrating photons in the 159 keV energy window [Fig. 2(d)]. Furthermore, the WEHR square holes are better matched to the detector element dimension, which probably improves the sensitivity relative to the MEHRS hexagonal holes. However, in C‐SPECT, there is an increase in the number of scattering components due to hole tailing and high‐energy γ‐rays. WEHR is also greatly affected by this factor because of its structure. However, MEHRS can reduce this impact. As such, it is likely that the sensitivity improved because WEHR contained many scattering components, as Ct.

Figure 3 shows that with C‐SPECT, visualization of line pairs as low as 3.0 mm was possible under the acquisition conditions, and an improved contrast resolution was observed. This can be understood from the perspective of frequency characteristics as well. The bar of 3.0 mm has a fundamental frequency of 1.52 cycle/cm for C‐SPECT_WEHR and 1.39 cycle/cm for A‐SPECT_LEHR based on the pixel size and Nyquist sampling. Figure 4 illustrates that C‐SPECT_WEHR shows a decrease in the fundamental frequency domain, indicating the limit of image decomposition. By contrast, A‐SPECT_LEHR showed constant signal intensity from 1.39 cycle/cm onward, indicating low contrast and difficult separation. Next, in the evaluation of SPECT, the evaluation of spatial resolution (Table 4) showed that with ^99m^Tc, the narrower the EW of C‐SPECT_WEHR, the higher the spatial resolution. This suggests that the narrowing of EW in C‐SPECT improves spatial resolution. For ^123^I, C‐SPECT_MEHRS had a lower resolution than C‐SPECT_WEHR and was equivalent to A‐SPECT_LMEGP. This is due to the geometrical arrangement of the collimator design. Compared with WEHR, MEHRS has a shorter hole length and a larger hole diameter; hence, the resolution is considered to have deteriorated. Moreover, regarding ASR, there was almost no distortion in the center under all imaging conditions of each radionuclide. However, distortions in the tangential direction on the outer periphery of the *x‐* and *y*‐axes deteriorated slightly in C‐SPECT when compared with A‐SPECT. This difference observed is likely because in C‐SPECT, the close distance between the radiation source and the detector led to an improved radial spatial resolution.

Next, with regard to the linearity of the radioactivity concentration, the slope of the regression line in Fig. 6 and Table 5 shows the sensitivity of the SPECT acquisition. With ^99m^Tc, A‐SPECT_LEHR showed the highest sensitivity in planar acquisition because of the collimator shape, whereas C‐SPECT_WEHR15 showed the highest sensitivity in SPECT. This can be attributed to the influence of SC in the DEW method. Because the effect of scattering in the Compton region is greater in C‐SPECT_WEHR15 than in A‐SPECT_LEHR, the SC set in this verification may have been insufficient to remove the scattered components in the main peak, resulting in an increase in both Ct and sensitivity. Therefore, in C‐SPECT_WEHR, it is useful to narrow the scatter correction SW to improve the sensitivity, but it is necessary to be careful that the scattered components do not increase. For ^123^I, A‐SPECT_LMEGP was the most sensitive, similar to the planar acquisition. However, C‐SPECT_MEHRS was more sensitive than C‐SPECT_WEHR and showed different results from that of the planar acquisition. The effect of SC can be considered in this case as well. As compared with MEHRS, WEHR is more greatly affected by scattering in the Compton and high‐energy regions. Therefore, removal of more scattering components than MEHRS through SC using the TEW method likely led to a low Ct and decreased the sensitivity of C‐SPECT_WEHR. Furthermore, by setting the SW for TEW scattering correction to 3% of the main peak, increase in sensitivity in C‐SPECT_WEHR is larger than that in C‐SPECT_MEHRS. Therefore, in C‐SPECT_WEHR, the scattered ray content greatly changes depending on the setting of the SW.

Figures 7 and 8 show the possibility of separating 8‐mm rods under high‐contrast conditions. This can be understood from the details in Fig. 8. Because smoothing processing was performed with a cutoff of 0.60 cycle/cm, equivalent to the fundamental frequency of an 8‐mm rod, the signal components that formed the 8‐mm rod were mainly low‐frequency. This is because the high‐frequency components beyond the fundamental frequency were removed. Therefore, with ^99m^Tc, the fact that C‐SPECT_WEHR showed a higher signal strength than A‐SPECT_LEHR in the high‐frequency region after 0.60 cycle/cm was not due to the noise component but rather to the edge component of contour formation. With ^123^I, in C‐SPECT_MEHRS, the signal strength was high in the high‐frequency range and was close to that of C‐SPECT_WEHR. This trend is the same as that of C‐SPECT_WEHR and shows the possibility to profile and separate the 8‐mm rod more than A‐SPECT. Furthermore, the tendency of ^123^I_SC3 of each collimator was close to that of ^123^I_SC7, indicating that the contrast resolution was retained.

In the homogeneity evaluation (Fig. 9), ^99m^Tc was similar under each condition but slightly improved in ^99m^Tc_SC3 of C‐SPECT_WEHR15. This finding does not contradict the sensitivity results in Fig. 6(a). With ^123^I, the findings do not contradict the sensitivity results shown in Fig. 6(b). The homogeneity was improved in ^123^I_SC3. The reason that the homogeneity was improved by the setting of ^123^I_SC3 is that C‐SPECT_MEHRS was able to acquire the primary photons with high accuracy rather than the increased scattered radiation (Table 5b). At the same time, C‐SPECT_WEHR and A‐SPECT_LMEGP showed improved homogeneity due to increased scattered radiation. It can be inferred from these results that C‐SPECT_MEHRS is suitable for ^123^I imaging because of improved homogeneity due to excellent scattered ray removal.

Our results further suggest that it is necessary to pay attention when conducting a direct comparison of each radionuclide because in clinical applications, the sealed concentrations differ for each radionuclide. Additionally, attention must be paid to the comparison of collimator design because there is a difference in the C‐SPECT and A‐SPECT system configurations. However, C‐SPECT showed superior energy resolution in each radionuclide when compared with A‐SPECT, leading to an improved contrast resolution. In addition, Peng et al reported a method for improving the crosstalk in the TEW method in SC with a CZT detector‐equipped device for the heart.^26^ In our results, the sensitivity and homogeneity were affected by the difference in the SC TEW method settings. However, by setting the SW for TEW scattering correction to 3% of the main peak in C‐SPECT, the contrast resolution is superior to that of A‐SPECT, imaging with similar homogeneity is possible, and improvement in quantification can be expected.

This study has some limitations. First, the availability of the evaluated radionuclides was limited. For MEHRS, in particular, the applicability of medium‐energy radionuclides ^111^In and ^177^Lu could be considered. However, given the characteristics of each radionuclide, the evaluation parameters will likely differ from those used in this study. Verification will be necessary with each imaging technique. Second, in this study, statistical noise was reduced in terms of system performance evaluation, but there is still a large influence of statistical noise in clinical practice. Therefore, it is necessary to verify each imaging process by including the noise components. Another limitation is related to the correction methods. As SPECT and CT are being used globally, attenuation correction of γ‐rays based on CT attenuation maps is considered useful.^27^, ^28^ SPECT images with corrections that improve positional resolution and reduce deterioration caused by collimator opening width and source distance combined with order subset expectation maximization are also clinically useful.^29^, ^30^ When these corrections are combined, the target noise components change, and setting of the optimal condition based on an understanding of the noise for each correction is required. Furthermore, it is necessary to verify the scatter correction factor in each imaging to apply a more accurate scatter correction.

## 5 CONCLUSION

We verified the system performance of C‐SPECT using ^99m^Tc and ^123^I. Each C‐SPECT system had superior energy resolution when compared with the A‐SPECT system and showed improved contrast resolution with planar and SPECT acquisition. In addition, with the newly developed MEHRS collimator, this study showed excellent scattered ray removal and high‐energy resolution on imaging with ^123^I formulation, demonstrating its clinical applicability. Furthermore, the verification results show that proper setting of the SW for TEW scatter correction improves the sensitivity and uniformity while maintaining contrast resolution. Clinical versatility can be enhanced by verifying each imaging technique in future studies.

## AUTHORS CONTRIBUTIONS

Toshimune Ito is the main author. Toshimune Ito and Masaaki shishido carried out the phantom study, data analysis, and drafted the manuscript. Masahisa Onoguchi, Koichi Okuda, Hajime Ichikawa and Takayuki Shibutani participated in its design and coordination and helped to draft the manuscript. Kozo Sato and Yohji Matsusaka revised manuscript critically. All authors read and approved the final manuscript.

## CONFLICT OF INTEREST

The authors have no relevant conflicts of interest to disclose.
